# Systematic Review and Meta‐Analysis of the Efficacy of BIIB080 (IONIS‐MAPTRx): The First Gene Therapy for Patients with Alzheimer’s Disease

**DOI:** 10.1002/alz.095608

**Published:** 2025-01-09

**Authors:** Abdalla Ahmed Mahmoud, Hamza Mohamed Ramadan

**Affiliations:** ^1^ College of Medicine, Arab Academy For Science, Technology and Maritime Transport, New Alamein, Matrouh Egypt; ^2^ Arab Academy For Science, Technology and Maritime Transport, El Alamein, matrouh Egypt

## Abstract

**Objective:**

This systematic review aims to evaluate the impact of tau synthesis reduction on tau biomarkers in patients receiving BIIB080 (IONIS‐MAPTRx) treatment for mild Alzheimer’s disease (AD).

**Background:**

Alzheimer’s disease is characterized by the accumulation of tau protein aggregates in the brain, leading to cognitive decline. Tau pathology, including abnormal phosphorylation and aggregation of tau protein, is closely associated with disease progression. BIIB080 is a novel therapeutic agent designed to target tau synthesis, potentially modifying the course of AD by reducing tau pathology.

**Methods:**

A comprehensive search was conducted across major scientific databases: Scopus, ScienceDirect, ClinicalKey, Cochrane, Clarivate, and PubMed for relevant studies and clinical trials. Inclusion criteria encompassed studies evaluating the effect of BIIB080 on tau biomarkers in patients with mild AD. Forty‐nine studies and two clinical trials were identified for screening.

**Results:**

Out of 102 evaluated participants, 46 with moderate AD were included; their mean age was 65.8 years (SD 5.70). BIIB080 demonstrated good tolerability and induced a dose‐dependent decrease in cerebrospinal fluid (CSF) total tau (t‐tau) and phosphorylated tau 181 (p‐tau181) throughout the mild AD stage. Significant reductions in t‐tau (56%; 95% CI, 50% to 62%) and p‐tau181 (51%; 95% CI, 38% to 63%) in CSF were observed in the two higher‐dose cohorts. This decline continued or was maintained during the long‐term extension phase with quarterly dosage. At week 25, tau positron emission tomography (PET) imaging showed less buildup in 13 subjects compared to placebo. By week 100, reductions from baseline were evident across all assessed regions (n = 12), with the most pronounced reductions observed in the temporal composite (‐0.71 standardized uptake value ratio; 95% CI, ‐1.40 to ‐0.02). A moderate correlation was noted between model‐predicted cumulative CSF drug exposure and tau PET change.

**Conclusion:**

BIIB080, as a tau synthesis reduction drug, demonstrated promising results on tau biomarkers in individuals with moderate AD, along with favorable tolerability. These findings suggest its potential as a therapeutic intervention to target tau pathology and alter the course of AD. Further research is warranted to establish its clinical effectiveness and long‐term safety profile.

